# Treatment shortening of drug-sensitive pulmonary tuberculosis using high-dose rifampicin for 3 months after culture conversion (Hi-DoRi-3): a study protocol for an open-label randomized clinical trial

**DOI:** 10.1186/s13063-022-06631-z

**Published:** 2022-08-17

**Authors:** Nakwon Kwak, Doosoo Jeon, Youngmok Park, Young Ae Kang, Kyung Jong Kim, Young Ran Kim, Byoung Soo Kwon, Yong-Soo Kwon, Hyung-Jun Kim, Jae Ho Lee, Ji Yeon Lee, Jung-Kyu Lee, Jeongha Mok, Minkyoung Cheon, Jiwon Park, Seokyung Hahn, Jae-Joon Yim

**Affiliations:** 1 Division of Pulmonary and Critical Care Medicine, Department of Internal Medicine, Seoul National University College of Medicine, Seoul, South Korea; 2 Division of Pulmonary and Critical Care Medicine, Department of Internal Medicine, Pusan National University Yangsan Hospital, Yangsan, South Korea; 3 Division of Pulmonary and Critical Care Medicine, Department of Internal Medicine, Yonsei University College of Medicine, Severance Hospital, Seoul, South Korea; 4 Department of R&D, Korean Institute of Tuberculosis, Cheongju, South Korea; 5 Clinical Research Section, International Tuberculosis Research Center, Seoul, South Korea; 6 Division of Pulmonary and Critical Care Medicine, Department of Internal Medicine, Seoul National University Bundang Hospital, Seongnam, South Korea; 7 Department of Internal Medicine, Chonnam National University Medical School, Chonnam National University Hospital, Gwangju, South Korea; 8 Division of Pulmonary and Critical Care Medicine, Department of Internal Medicine, National Medical Center, Seoul, South Korea; 9 Division of Pulmonary and Critical Care Medicine, Department of Internal Medicine, Seoul National University Boramae Medical Center, Seoul, South Korea; 10 Division of Pulmonology, Allergy and Critical Care Medicine, Department of Internal Medicine, Pusan National University Hospital, Busan, South Korea; 11 Medical Research Collaborating Center, Seoul National University Hospital, Seoul, South Korea; 12 Department of Human Systems Medicine, Seoul National University College of Medicine, Seoul, South Korea

**Keywords:** Tuberculosis, Shorter regimen, Rifampicin

## Abstract

**Background:**

The standard treatment regimen for drug-sensitive tuberculosis (TB), comprising four companion drugs, requires a minimum duration of 6 months, and this lengthy treatment leads to poor adherence and increased toxicity. To improve rates of adherence, reduce adverse events, and lower costs, a simplified and shortened treatment regimen is warranted.

**Methods:**

This study is a multicenter, open-label randomized clinical trial of non-inferiority design that compares a new regimen with the conventional regimen for drug-sensitive pulmonary TB. The investigational group will use a regimen of high-dose rifampicin (30 mg/kg/day) with isoniazid and pyrazinamide, and the treatment will be maintained for 12 weeks after the achievement of negative conversion of sputum culture. The control group will be treated for 6 months with a World Health Organization-endorsed regimen consisting of isoniazid, rifampicin (10 mg/kg/day), ethambutol, and pyrazinamide. The primary endpoint is the proportion of unfavorable outcomes at 18 months after randomization. Secondary outcomes include time to unfavorable treatment outcome, time to culture conversion on liquid medium, treatment success rate at the end of treatment, proportion of recurrence at 18 months after randomization, time to recurrence after treatment completion, and adverse events of grade 3 or higher during the treatment. We predict a 10% unfavorable outcome for the control group, and 0% difference from the investigational group. Based on 80% verification power and a 2.5% one-sided significance level for a non-inferiority margin of 6%, 393 participants per group are required. Considering the 15% dropout rate, a total of 926 participants (463 in each group) will be recruited.

**Discussion:**

This study will inform on the feasibility of the treatment regimen using high-dose rifampicin with a shortened and individualized treatment duration for pulmonary TB.

**Trial registration:**

ClinicalTrials.gov NCT04485156. Registered on July 24, 2020.

**Supplementary Information:**

The online version contains supplementary material available at 10.1186/s13063-022-06631-z.

## Background

Tuberculosis (TB) remains one of the most important infectious diseases. Globally, around 5.8 million people fell ill with TB and 1.5 million people died from TB in 2021. Moreover, ~ 25% of the world’s population has latent TB infection [1]. In South Korea, 22,904 TB cases were reported with a notification rate of 44.6 per 100,000 population in 2021 [2]. Although TB remains a global health concern, TB can be cured with medical treatment, and the success rate for drug-sensitive TB can reach 85% [1].

The standard treatment regimen for drug-sensitive TB, which was introduced in the 1980s, consists of four drugs: isoniazid, rifampicin, pyrazinamide, and ethambutol [3]. While this regimen has proven to be effective, it typically requires a minimum 6-month treatment duration, irrespective of disease severity or extent [4]. This lengthy treatment leads to poor adherence and increased toxicity [5]. To improve rates of adherence, reduce adverse events, and lower costs, several clinical trials have investigated approaches that aim to shorten the treatment duration in TB patients; however, these studies have failed to prove non-inferiority to the standard 6-month regimen, with the exception of a 4-month regimen based on rifapentine and moxifloxacin [6–9].

To shorten the treatment duration, the sterilizing activity of anti-TB drugs should be taken into consideration, and rifampicin is a cornerstone drug for the maintenance of sterilizing activity. Rifampicin, a derivate of rifamycin, is effective against *Mycobacterium tuberculosis* populations in a dormant state, as well as those undergoing rapid metabolism [3, 10]. Therefore, rifampicin has the potential to shorten treatment duration to less than 6 months [10]. The area under the concentration–time curve and peak concentration of rifampicin showed a more than proportional increase with a dose change from 10 to 35 mg/kg/day [11, 12]. The higher exposure to rifampicin with the dose increment led to a more rapid fall in bacterial loads [11, 12]. Despite these favorable dose–response relationships of rifampicin, the maximal recommended dose is limited to 10 mg/kg/day or 600 mg/day, owing to the concern of adverse events [13]. However, the use of rifampicin at doses higher than the recommended dose did not cause additional serious adverse events [14, 15]. The number of serious adverse events, including hepatotoxicity, was independent of rifampicin dose in two recent randomized controlled trials [14, 15]. According to real clinical practice data from a Dutch cohort, 6–12 months of high-dose rifampicin therapy was safe and well-tolerated in 99% of patients [16].

Based on the literature, we propose a simplified and shortened treatment strategy using high-dose rifampicin. The effectiveness, safety, and tolerability of a three-drug therapy using high-dose rifampicin with isoniazid and pyrazinamide will be investigated in comparison with the standard 6-month treatment for TB. The protocol of a phase 3, multicenter, randomized, open-label non-inferiority clinical trial, entitled “Treatment shortening of drug-sensitive pulmonary TB using high-dose rifampicin for 3 months after culture conversion (Hi-DoRi-3)” is presented here.

## Methods/design

### Study design

This investigator-initiated study is a prospective, multicenter, randomized, open-label, two-arm non-inferiority clinical trial. Participants with drug-sensitive pulmonary TB will be randomly assigned to one of two groups at a 1:1 ratio, and the study will be terminated 18 months after randomization.

### Setting

Participants will be recruited from eight referral hospitals located in South Korea: (1) Seoul National University Hospital, (2) National Medical Center, (3) Pusan National University Hospital, (4) Seoul National University Bundang Hospital, (5) Seoul Metropolitan Government-Seoul National University Boramae Medical Center, (6) Severance Hospital, (7) Pusan National University Yangsan Hospital, and (8) Chonnam National University Hospital.

### Control group

Participants randomized to the control group will receive standard treatment in accordance with the Korean guidelines for TB [17] and the World Health Organization guidelines [4]. Consequently, patients in the control group will be administered isoniazid, rifampicin, pyrazinamide, and ethambutol for 2 months, followed by isoniazid, rifampicin, (and ethambutol) for 4 months, comprising a total treatment period of 6 months (Table 1). Although temporary use of secondary anti-TB drugs is permitted in the case of adverse events such as liver toxicity, the total duration of use should be less than 4 weeks. If rifampicin is discontinued because of adverse events, the period of discontinuation must also be less than 4 weeks.

**Table 1:** Dose and schedule of anti-tuberculous drugs for the control group

Drug name	Capacity			Administration method
	Per bodyweight	Recommended dose	Maximum dose	
Isoniazid	5 mg/kg	300 mg/day	300 mg/day	once a day before meal
Rifampicin	10 mg/kg	450 mg (< 50 kg) 600 mg (≥ 50 kg)	600 mg/day	once a day before meal
Pyrazinamide	20–30 mg/kg	1000 mg (< 50 kg) 1500 mg (50–70 kg) 2000 mg (> 70 kg)		once a day after meal
Ethambutol	15–20 mg/kg	800 mg (< 60 kg) 1200 mg (60–80 kg) 1600 mg (> 80 kg)		once a day after meal

### Investigational group

**Fig. 1:**
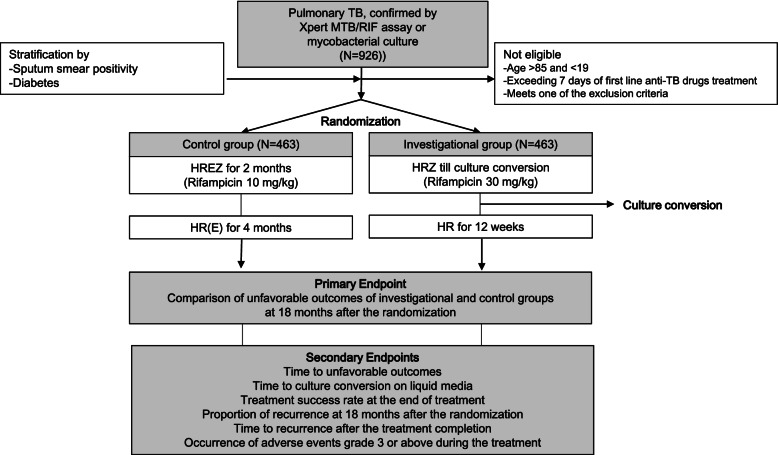
Study overview

Participants randomized to the investigational group will be treated with high-dose rifampicin (body weight, dose: < 44 kg, 1200 mg/day; 45–54 kg, 1500 mg/day; 55–64 kg, 1800 mg/day; 65–74 kg, 2100 mg/day; > 75 kg, 2400 mg/day), isoniazid (300 mg/day), and pyrazinamide (body weight, dose: < 50 kg, 1000 mg/day; 50–70 kg, 1500 mg/day; > 70 kg, 2000 mg/day) until culture conversion (liquid medium) is confirmed (Table 2). High-dose rifampicin and isoniazid will be administered until 12 weeks after the date of sputum culture negative conversion. The dosage of rifampicin will be maintained throughout the study duration. Ethambutol will not be included in the initial regimen to reduce the number of pills. However, in the instance that isoniazid or pyrazinamide is discontinued prior to the end of treatment, ethambutol may be added to the treatment regimen if the investigator decides that reinforcement of the drug is necessary. Although temporary use of secondary anti-TB drugs is permitted in the case of adverse events such as liver toxicity, the total duration of use should be less than 4 weeks. If rifampicin is discontinued because of adverse events, the period of discontinuation must also be less than 4 weeks. The study overview is provided in Fig. 1.

**Table 2:** Dose and schedule of anti-tuberculous drugs for the investigational group

Drug name	Dose (body weight)	Administration method
Isoniazid	300 mg/day	once a day before meal
Rifampicin	1200 mg/day (< 44 kg) 1500 mg/day (45–54 kg) 1800 mg/day (55–64 kg) 2100 mg/day (65–74 kg) 2400 mg/day (> 75 kg)	once a day before meal
Pyrazinamide	1000 mg/day (< 50 kg) 1500 mg/day (50–70 kg) 2000 mg/day (> 70 kg)	once a day after meal

### Study population

A total of 926 participants with drug-sensitive pulmonary TB will be enrolled in the study (463 participants per group). Adult participants aged between 19–85 years whose sputum (or bronchoscopic specimen) is positive for Xpert MTB/RIF assay or *M. tuberculosis* culture, and who have been taking any TB medication for no more than 7 days at the time of enrollment, will be included. The exclusion criteria are as follows: (1) rifampicin resistance confirmed by Xpert MTB/RIF assay; (2) drug resistance to isoniazid, rifampicin, or pyrazinamide, confirmed by a drug-susceptibility test; (3) human immunodeficiency virus infection; (4) malignancy needing anti-cancer chemotherapy; (5) chronic hepatitis or liver cirrhosis; (6) any contraindications for the study drugs (hypersensitivity to the study drugs, biliary obstruction, galactose intolerance, Lapp lactase deficiency, or glucose-galactose malabsorption); (7) current use of drugs, such as indinavir, saquinavir, nelfinavir, amprenavir, praziquantel, or atovaquone, or any other drugs contraindicated in combination with study drugs, within 1 day before study enrollment, or expected to be used within the following 28 days; and (8) pregnancy, breastfeeding, or pregnancy planned within the following 6 months.

### Assignment of interventions: allocation

#### Sequence generation

The randomization table will be generated and will be reproducible via SAS 9.4 software, using the stratified block randomization method with a stratification factor of (1) acid-fast bacilli (AFB) smear positivity and (2) presence of diabetes. The Medical Research Collaborating Center (MRCC) of the Seoul National University Hospital will operate a web-based randomization system remotely.

#### Allocation concealment mechanism

It is an open-label trial. Both participants and treating physicians will be aware of allocation information.

#### Implementation

Participants will be provided with a detailed explanation of the purpose, method, and procedure for this study, and written consent for participation in the clinical trial will be obtained. Prior to randomization, participant demographic information, medical history, and physical examination results will be collected, including results of the Xpert MTB/RIF assay, AFB smear (including *M. tuberculosis* culture), chest X-ray, blood test, and urine (or serum) human chorionic gonadotropin (hCG; for women of childbearing age) test. After completing the consent procedure, participants who meet the inclusion criteria will be randomly assigned to the investigational or control group at a ratio of 1:1 in week 0.

### Treatment visits

Following enrollment, participants of each group will visit the hospital every 2 weeks until 8 weeks after enrollment. Subsequently, visits will be made every 4 weeks until the end of treatment. The tests performed at each visit will include the following: physical examination, AFB smear and culture using a liquid medium, and blood test. For women of childbearing age, a urine or blood hCG analysis will be performed. Simple chest X-ray will be performed every 4 weeks. Participants who cannot produce sputum will be encouraged to expectorate sputum. If the participants cannot produce sputum, it will not be deemed as protocol violation.

### Follow-up

After the end of treatment, visits will be made every 3 months up until 18 months after the enrollment date. The physical examination, AFB smear and culture, and chest X-ray tests will be performed at each visit. The study timeline is shown in Tables 3 and 4.

**Table 3:** Time line for control group

Type	Screening^a^	Baseline^a^	Treatment				End of treatment (EOT)^h^	Follow-up				End of study (EOS)
Visit name	Screening	D0	W2	W4	W6	W8	W12 ~ (every 4 W)	EOT + 3 M	EOT + 6 M	EOT + 9 M	EOT + 12M^i^	M18^i^
Weeks (w)	− 14 ~ 0 days	0	2 W	4 W	6 W	8 W	12 W/16 W/20 W/24 W /28 W/32 W	EOT + 13 W	EOT + 26 W	EOT + 39 W	EOT + 52 W	78 W
Visit window	n-a	n-a	± 4 days	± 4 days	± 4 days	± 4 days	± 2 weeks	± 6 weeks	± 6 weeks	± 6 weeks	± 6 weeks	± 6 weeks
Consent	O											
Randomization		O										
Medical history	O											
Physical exam	O	O	O	O	O	O	O	O	O	O	O	O
Xpert MTB/RIF assay	O^e^											
Sputum AFB smear	O^e^	O^e^	O	O	O	O	O	O	O	O	O	O
TB culture (liquid)	O^e^	O^e^	O	O	O	O	O	O	O	O	O	O
DST^b^	With first/reverted cultured MTB											
Chest X-ray	O^e^	O^e^		O		O	O	O	O	O	O	O
Chemistry^c^	O^e^	O^e^	O	O	O	O	O^h^					
Complete blood count	O^e^	O^e^	O	O	O	O	O^h^					
HIV	O^e^											
Urine (or blood) HCG^d^	O	O	O	O	O	O	O^h^					
HBV/HCV^f^		O^e^										
Compliance of drug intake			O	O	O	O	O^h^					
Adverse drug reaction			O	O	O	O	O^h^	O				
Other medication^g^	O		O	O	O	O	O^h^					

^a^ It can be performed with the baseline visit if all screening results can be checked on the day

^b^ Drug susceptibility test for isoniazid, rifampicin, ethambutol, pyrazinamide, streptomycin, kanamycin, amikacin, capreomycin, ofloxacin, levofloxacin, moxifloxacin, prothionamide, cycloserine, rifabutin, and para-aminosalicylic acid (Omitted for the patients whose results are already reported)

^c^ Test items according to the standard treatment guidelines (refer to 6. Study procedures)

^d^ Performed for women of childbearing age tested within 7 days

^e^ The existing test results can be extracted and collected without additional testing if the previous test was performed within 4 weeks (based on the report day for the TB culture)

^f^ Since HBsAg and Anti-HCVAb are not excluded even if they are positive, drugs can be administered even before the results are reported

^g^ Confirmation of concomitant drugs, especially immunosuppressants including oral steroids

^h^ The EOT date is determined according to standard of care guidelines. A visit every 4 weeks until EOT and every 3 months after EOT. The test is not performed after EOT

^i^ If the 78-week visit (EOS) after randomization arrives earlier than the EOT + 9 M visit, the EOT + 9 M visit is omitted, and the EOS visit proceeds

**Table 4:** Timeline for the investigational group

Type	Screening^a^	Baseline^a^	Treatment				End of treatment (EOT)^h^	Follow-up				End of study (EOS)
Visit name	Screening	D0	W2	W4	W6	W8	W12 ~ (Every 4 W)	EOT + 3 M	EOT + 6 M	EOT + 9 M	EOT + 12M^i^	M18^i^
Weeks (w)	− 14 ~ 0 days	0	2 W	4 W	6 W	8 W	12 W/16 W/20 W/24 W /28 W/32 W	EOT + 13 W	EOT + 26 W	EOT + 39 W	EOT + 52 W	78 W
Visit window	n-a	n-a	± 4 days	± 4 days	± 4 days	± 4 days	± 2 weeks	± 6 weeks	± 6 weeks	± 6 weeks	± 6 weeks	± 6 weeks
Consent	O											
Randomization		O										
Medical history	O											
Physical exam	O	O	O	O	O	O	O	O	O	O	O	O
Xpert MTB/RIF assay	O^e^											
Sputum AFB smear	O^e^	O^e^	O	O	O	O	O	O	O	O	O	O
TB culture (liquid)	O^e^	O^e^	O	O	O	O	O	O	O	O	O	O
DST^b^	With first/reverted cultured MTB											
Chest X-ray	O^e^	O^e^		O		O	O	O	O	O	O	O
Chemistry^c^	O^e^	O^e^	O	O	O	O	O^h^					
Complete blood count	O^e^	O^e^	O	O	O	O	O^h^					
HIV	O^e^											
Urine (or blood) HCG^d^	O	O	O	O	O	O	O^h^					
HBV/HCV^f^		O^e^										
Compliance of drug intake			O	O	O	O	O^h^					
Adverse drug reaction			O	O	O	O	O^h^	O				
Other medication^g^	O		O	O	O	O	O^h^					

^a^ It can be performed with the baseline visit if all screening results can be checked on the day

^b^ Drug susceptibility test for isoniazid, rifampicin, ethambutol, pyrazinamide, streptomycin, kanamycin, amikacin, capreomycin, ofloxacin, levofloxacin, moxifloxacin, prothionamide, cycloserine, rifabutin, and para-aminosalicylic acid (Omitted for the patients whose results are already reported)

^c^ Test items according to the standard treatment guidelines (refer to 6. Study procedures)

^d^ Performed for women of childbearing age tested within 7 days

^e^ The existing test results can be extracted and collected without additional testing if the previous test was performed within 4 weeks (based on the report day for the TB culture)

^f^ Since HBsAg and Anti-HCVAb are not excluded even if they are positive, drugs can be administered even before the results are reported

^g^ Confirmation of concomitant drugs, especially immunosuppressants including oral steroids

^h^ The date of the end of treatment is determined according to the time of culture conversion to negative. A visit every 4 weeks until EOT and every 3 months after EOT. The test is not performed after EOT

^i^ If the 78-week visit (EOS) after randomization arrives earlier than the EOT + 12 M visit, the EOT + 12 M visit is omitted, and the EOS visit proceeds

### Definitions

Culture of *M. tuberculosis* will be considered to be converted to negative when two or more consecutive cultures, taken at different visits, are found to be negative in liquid medium. When a participant cannot produce sputum after the first negative culture sputum, they will be considered as having achieved culture conversion. The submission date of the first confirmed negative sputum culture will be regarded as the date of culture conversion. If *M. tuberculosis* is found to be re-cultured two or more times following a negative culture conversion, this will be termed culture reversion [18]. Treatment success is defined as achieving negative culture conversion and maintaining negative cultures till the end of treatment.

The sum of treatment failure, recurrence, death during treatment (except for accidents and suicide), loss to follow-up, and withdrawal from the study will be designated as “unfavorable outcomes.” Treatment failure will be defined as participants who fail to achieve culture conversion during treatment period, or as participants using second-line anti-TB drugs for more than 4 weeks. Recurrence will be assigned to participants that indicate the presence of *M. tuberculosis* in sputum cultures for two or more times after the end of treatment.

Adverse events will be defined when undesirable medical events have occurred in participants, irrespective of a causal relationship with the treatment [19]. When adverse events result in death, life-threatening conditions, patient hospitalization or prolongation of existing hospitalization, persistent or significant disability or incapacity, or a congenital anomaly or birth defect, the events will be classified as serious adverse events. The severity of each adverse event will be assessed based on Common Terminology Criteria for Adverse Events (CTCAE) version 5.0 [20].

### Study outcomes

The primary outcome is the proportion of unfavorable outcomes at 18 months after randomization.

Secondary outcomes include time to unfavorable treatment outcomes, time to culture conversion on liquid medium, treatment success rate at the end of treatment, proportion of recurrence at 18 months after randomization, time to recurrence after treatment completion, and adverse events of grade 3 or higher during treatment.

### Sample size calculation

The objective of this study is to determine whether the new regimen is non-inferior to the existing standard treatment. The primary outcome is an unfavorable treatment outcome at 18 months after randomization. Based on the existing study [21], a 10% unfavorable treatment outcome for the standard treatment (control group) and a 0% difference from the investigational group is assumed. To obtain a study with 80% power and a 2.5% one-sided significance level for the non-inferiority recognition limit of 6%, each group will contain 393 participants. Assuming a 15% dropout rate [22], the total number of study participants will be 926, resulting in 463 participants in each group. In the occurrence of participant withdrawal within 7 days of randomization, replacement participants may be recruited.

### Statistical analysis

The results of this trial for outcomes will be analyzed based on intention-to-treat (ITT), modified ITT (mITT), and per protocol (PP) approaches, with a primary consideration for mITT results. The ITT group will include all participants who are randomized after satisfying eligibility criteria. The mITT group will include participants who are randomized and who have received the study drugs at least once. The PP group will include participants in the mITT group who have completed the standard treatment for at least 20 of the 24 weeks for the control group or those who have completed the planned treatment for at least 10 of the 12 weeks for the investigational group. A safety analysis will be performed based on the safety group, which will include participants who have been administered with at least one dose of the investigational drugs.

For the primary outcome analysis, the frequency of occurrence of unfavorable outcomes in the control and investigational groups will be analyzed, and the incidence rate of unfavorable outcomes (${\widehat{P}}_{T}, {\widehat{P}}_{C}$) and the 95% confidence interval will be estimated. In this non-inferiority test, if the upper limit of the two-sided 95% confidence interval of the difference in incidence of unfavorable outcomes at 18 months between the investigational and control groups (${\widehat{P}}_{T}-{\widehat{P}}_{C}$) is less than 6%, it will be concluded that the investigational group is not inferior to the control group.

For the analysis of time to unfavorable outcome, time to culture conversion, and time to recurrence, the median time of each group will be estimated using the Kaplan–Meier method and will be compared with the log-rank test. Treatment success rate and recurrent rate at the end of treatment will be compared with the chi-square test or Fisher’s exact test between the two groups.

Safety will be assessed based on the occurrence of adverse events during the clinical trial period. The frequency and proportion of all adverse events, serious adverse events, and adverse events of grade 3 or higher will be summarized and compared using the chi-square test or Fisher’s exact test.

### Data collection and management

This study will use a web-based electronic case report form (e-CRF) with Research Electronic Data Capture (REDCap) constructed by the MRCC in collaboration with an outsourced contractor. The e-CRF designed for this study will use dummy variables for user acceptance testing to confirm its validity. During the study, medical personnel not participating in the study will monitor the trial. These personnel will visit study sites to monitor all aspects of the trial, including adherence to the protocol in accordance with Good Clinical Practice, study participant protection, and data accuracy of the study.

### Supervision of the trial

A Data and Safety Monitoring Board (DSMB), composed of two respiratory specialists who have experience treating TB patients and one statistician from another institute with no conflict of interests, will review data every 3 months during the trial. The DSMB could provide recommendations for protocol changes (including advice on continuation or cessation) based on the results.

### Confidentiality

The collection and operation of participant personal information in this study is limited to the data necessary for the efficacy, safety, and tolerability testing of the study drug. Data will be carefully collected and operated to comply with relevant data confidentiality laws and regulations and to ensure confidentiality. Paper files containing participant data (including personally identifiable information and copies of signed consent forms) will be securely stored in a locked office on site in locked filing cabinets. Digital files containing participant data will be stored as password-protected files on university-maintained servers. Access to study files will be restricted to authorized personnel only. The items in the present study protocol comply with the Standard Protocol Items: Recommendations for Interventional Trials (SPIRIT) checklist (see Additional file 1).

### Provisions for post-trial care

Participants with recurrence will receive standard TB treatment in accordance with guidelines.

### Dissemination

Trial results will be communicated to the scientific community via journals and national and international conferences.

## Discussion

The Hi-DoRi-3 is a phase 3 open-label, multicenter, randomized, non-inferiority clinical trial that aims to investigate the effectiveness of a high-dose rifampicin-containing regimen for the treatment of drug-sensitive TB. In this study, the effectiveness and safety of a regimen with a shortened and individualized treatment duration will be compared with the standard 6-month regimen for TB treatment. The three-drug intervention regimen will include high-dose rifampicin (30 mg/kg) with isoniazid and pyrazinamide, which will be administered until culture conversion, followed by 12-week administration of high-dose rifampicin and isoniazid.

In South Korea, the treatment success rate of TB is ~ 90%. While less than 1% of TB patients could not be cured, 5.3–25.2% of TB patients were lost to follow-up [23]. The standard 6-month regimen, comprising four drugs, has remained unchanged for almost 40 years [3]. To enhance compliance and adherence to TB treatment, simple, less toxic, and shorter regimens are needed. With the aim to shorten treatment duration in TB patients, 4-month fluoroquinolone-containing regimens were compared with the standard treatment [7–9]; however, non-inferiority of these regimens was not proven.

For the last ~ 40 years, recommendations for the maximal dose of rifampicin have been confined to 10 mg/kg or 600 mg per day [4, 17]. The peak concentration after ingestion of the currently recommended dose of rifampicin is considered to be well above the minimum inhibitory concentration of *M. tuberculosis* [13]. However, this dose is at the lower end of the dose–response curve, and the drug concentration in *M. tuberculosis* and at the site of infection has not been considered [13]. Increased rifampicin exposure led to higher bactericidal and sterilizing activity for TB according to several in vitro and in vivo studies [24–26]. In human studies, higher doses of rifampicin shortened the time to sputum culture conversion compared with the conventional rifampicin dose [12, 14, 27]. The rate of serious adverse events in patients receiving higher doses of rifampicin was comparable to that in patients receiving the conventional dose [16].

When rifapentine, a cyclopentyl derivate of rifampicin, was administered twice weekly with moxifloxacin for 2 months, followed by 2 months of moxifloxacin, ethambutol, rifampicin, and pyrazinamide, ~ 20% of patients had unfavorable outcomes, and this strategy was proven to be inferior to the standard regimen [8]. Fortunately, a recent study showed that daily administration of 1200 mg of rifapentine with moxifloxacin, isoniazid, and pyrazinamide could shorten the treatment duration by up to 4 months without non-inferiority [6]. In our trial, high-dose rifampicin, which is more affordable than rifapentine [28], will be tested as a means to shorten treatment duration. In addition, the total duration of treatment will be individualized according to the timing of culture conversion, which will reflect each patient’s disease severity and treatment response.

In conclusion, our study will inform on the feasibility of a treatment regimen using high-dose rifampicin with a shortened and individualized treatment duration for pulmonary TB.

### Trial status

Recruitment began at the first site in November 2020 and is expected to be completed by August 2025.

## Supplementary Information

**Additional file 1.** SPIRIT checklist

## Data Availability

The results of this trial will be disseminated only by presentation at academic meetings or publication in academic journals.

## Abbreviations

AFB: Acid-fast bacilli
CTCAE: Common Terminology Criteria for Adverse Event
e-CRF: Electronic case report form
DSMB: Data and Safety Monitoring Board
hCG: Human chorionic gonadotropin
mITT: Modified intention-to-treat
MRCC: Medical Research Collaborating Center
PP: Per protocol
REDCAP: Research Electronic Data Capture
TB: Tuberculosis
